# Optimization based automated curation of metabolic reconstructions

**DOI:** 10.1186/1471-2105-8-212

**Published:** 2007-06-20

**Authors:** Vinay Satish Kumar, Madhukar S Dasika, Costas D Maranas

**Affiliations:** 1 Department of Industrial and Manufacturing Engineering, The Pennsylvania State University, University Park, PA 16802, USA; 2 Department of Chemical Engineering, The Pennsylvania State University, University Park, PA 16802, USA

## Abstract

**Background:**

Currently, there exists tens of different microbial and eukaryotic metabolic reconstructions (e.g., *Escherichia coli, Saccharomyces cerevisiae*, *Bacillus subtilis*) with many more under development. All of these reconstructions are inherently incomplete with some functionalities missing due to the lack of experimental and/or homology information. A key challenge in the automated generation of genome-scale reconstructions is the elucidation of these gaps and the subsequent generation of hypotheses to bridge them.

**Results:**

In this work, an optimization based procedure is proposed to identify and eliminate network gaps in these reconstructions. First we identify the metabolites in the metabolic network reconstruction which cannot be produced under any uptake conditions and subsequently we identify the reactions from a customized multi-organism database that restores the connectivity of these metabolites to the parent network using four mechanisms. This connectivity restoration is hypothesized to take place through four mechanisms: a) reversing the directionality of one or more reactions in the existing model, b) adding reaction from another organism to provide functionality absent in the existing model, c) adding external transport mechanisms to allow for importation of metabolites in the existing model and d) restore flow by adding intracellular transport reactions in multi-compartment models. We demonstrate this procedure for the genome- scale reconstruction of *Escherichia coli *and also *Saccharomyces cerevisiae *wherein compartmentalization of intra-cellular reactions results in a more complex topology of the metabolic network. We determine that about 10% of metabolites in *E. coli *and 30% of metabolites in *S. cerevisiae *cannot carry any flux. Interestingly, the dominant flow restoration mechanism is directionality reversals of existing reactions in the respective models.

**Conclusion:**

We have proposed systematic methods to identify and fill gaps in genome-scale metabolic reconstructions. The identified gaps can be filled both by making modifications in the existing model and by adding missing reactions by reconciling multi-organism databases of reactions with existing genome-scale models. Computational results provide a list of hypotheses to be queried further and tested experimentally.

## Background

The genome of several microorganisms has been completely sequenced and annotated in the past decade [1-4]. This information has aided the metabolic reconstructions of several microbial and eukaryotic organisms using experimental evidence and bioinformatics based techniques providing single compartment (e.g., *Escherichia coli *[5]) and multi-compartment models (e.g., *Saccharomyces cerevisiae *[6]). All of these reconstructions are inherently incomplete with some functionalities missing due to the lack of experimental and/or homology information. These missing reaction steps may lead to the prediction of erroneous genetic interventions for a targeted overproduction or the elucidation of misleading organizational principles and properties of the metabolic network. A key challenge in the automated generation of genome-scale reconstructions is the elucidation of these gaps and the subsequent generation of hypotheses to bridge them. This challenge has already been recognized and a number of computational approaches have been under development to resolve these discrepancies [7-11].

Most of the aforementioned efforts are based on the use of sequence homology to uncover missing genes. Specifically, sequence homology is used to pinpoint genes in related species that have significant similarity with an unassigned ORF of the curated microorganism [12]. Green et al formalized and further extended this concept by introducing a method that identified missing enzymes in a metabolic network using sequence homology related metrics within a Bayesian framework [11]. Alternatively, non-homology based reconstructions have been implemented by identifying candidate genes by measuring the similarity with metrics such as mRNA co expression data [8] and phylogenetic profiles [10] while also taking into account the local structure of the existing partially reconstructed metabolic networks. A recent advancement in this direction uses multiple types of association evidence including clustering of genes on the chromosome and protein fusion events in addition to phylogenetic profiles [9]. All methods described above postulate a set of candidate genes and then evaluate the likelihood that any of these candidate genes is present in the microorganism's metabolic network of interest using a variety of scoring metrics. In addition to these approaches, various genomic context analyses have also been used to identify missing metabolic genes [7,13-16]. Specifically, a recent study exploits the availability of highly curated metabolic networks to hypothesize gene reaction interactions in less characterized organisms [16]. These aforementioned methods predict missing enzymes in the metabolic network by conducting sequence based comparisons of entire genomes and inferring possible metabolic functions across different microorganisms.

**Figure 1:**
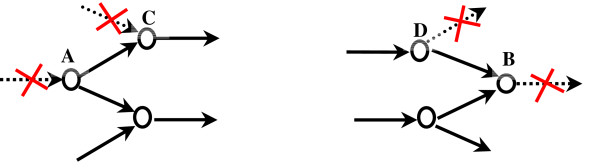
**Characterization of problem metabolites inmetabolic networks**. Metabolite A is defined as a root no-production metabolite because there is no-production or transport mechanism for it in the network. Metabolite C is a downstream no-production metabolite because, despite there being a reaction that produces it, it can carry no flux as A cannot be produced in the network. Equivalently, B and D are defined as root and downstream no-consumption metabolites.

Alternatively, a recent systems based computational approach identifies the location of missing metabolic functions in the *E. coli *iJR904 model by pinpointing discrepancies between *in silico *model predictions and known *in vivo *growth phenotypes [17]. Subsequently, an optimization based algorithm is used to resolve these discrepancies by searching for missing metabolic functions from a candidate database of reactions. In this paper instead, we pinpoint metabolites that cannot carry any flux through them and subsequently generate hypotheses to restore connectivity. To this end, we introduce an optimization based procedure (GapFind) to first identify such gaps in both single and multi-compartment metabolic networks and subsequently using an optimization based procedure (GapFill) restore their connectivity using separate pathology resolving hypotheses. In contrast to the previous methods which fill gaps only by identifying missing enzymes [8-11,17] or adding transport reactions [17], we also explore whether these gaps can be filled by making intra model modifications. Figure 1 pictorially illustrates how such gaps arise in metabolic reconstructions and introduces the definitions proposed in this paper to precisely describe these pathologies.

Gaps in metabolic reconstructions are manifested as (i) metabolites which cannot be produced by any of the reactions or imported through any of the available uptake pathways in the model; or (ii) metabolites that are not consumed by any of the reactions in the network or exported based on any existing secretion pathways. We refer to these metabolites as *root no-production *(e.g., metabolite A) and *root no-consumption *metabolites (e.g., metabolite B) respectively. At steady-state conditions no flow can pass through them due to the incomplete connectivity with the rest of the network. Clearly, such pathologies are not physiologically relevant and thus must be caused by omission and/or errors in the model reconstruction process. Notably, the lack of flow in root no-production metabolites and root no-consumption metabolites is propagated downstream/upstream respectively giving rise to additional metabolites that cannot carry any flow. We refer to these metabolites that are indirectly prevented from carrying flow as *downstream no-production *(e.g., metabolite C) metabolites and *upstream no-consumption *metabolites (e.g., metabolite D) respectively. It is important to note that by restoring connectivity for the root problem metabolites all upstream/downstream metabolites are also automatically fixed. We concentrate on resolving only no-production metabolites in the case of cytosolic metabolites. In the case of non-cytosolic (i.e., present in internal compartments) metabolites, we identify mechanisms to resolve both no-production and no-consumption metabolites.

**Figure 2:**
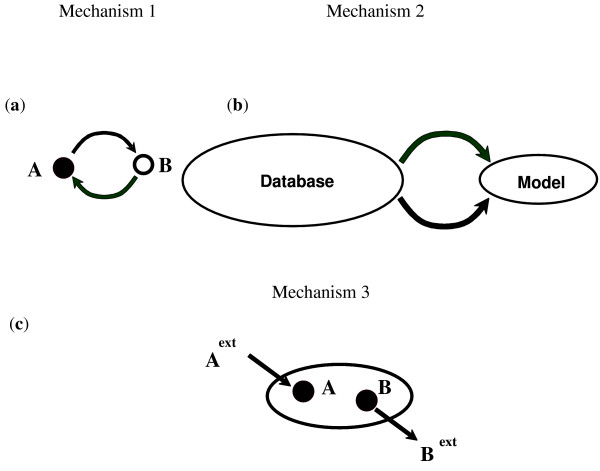
**Connectivity restoration mechanisms for problem metabolites**. In (a), reversing the directionality of a reaction restores the connectivity of metabolite (A) to the network. In (b) reactions are added from the external database to the model organism in order to enable production and consumption of no-production and no-consumption metabolites respectively. In (c), the production of no-production metabolite A is enabled by adding an external uptake pathway, the consumption of no-consumption metabolite B is enabled by adding an external secretion pathway.

**Figure 3:**
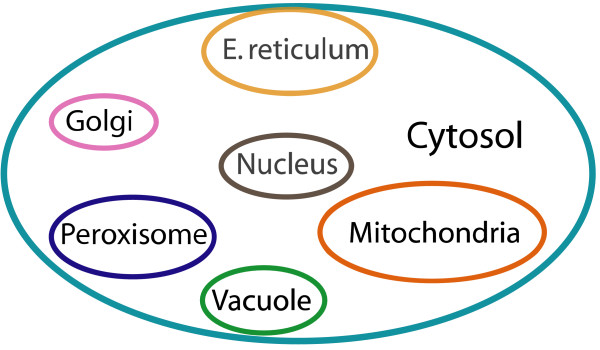
**The multi-compartment model of *Saccharomyces cerevisiae***. All compartments interact only with the cytosol. Only the cytosol interacts with the extracellular space.

For single compartment metabolic networks (where we have only cytosolic metabolites), we postulate three separate mechanisms for fixing no-production metabolites (see also Figure 2). We explore whether (i) reversing the directionality of existing reactions in the model (**Mechanism 1**), (ii) adding new reactions from a multi-species database (e.g., MetaCyc [18]) (**Mechanism 2**) or finally (iii) allowing for the direct importation of the problem metabolite restores flow into the no-production metabolite (**Mechanism 3**). For multi-compartment models, (e.g., *S. cerevisiae*) (Figure 3) we treat gaps in the cytosol differently than gaps in compartments (e.g., mitochondria, peroxisome etc). For cytosolic no-production metabolites, in addition to the three connectivity restoration mechanisms proposed for single compartment models, we additionally explore whether they can be fixed by adding intracellular transport reactions between compartments and the cytosol (**Mechanism 4**). For non-cytosolic problem metabolites, present in internal compartments, direct importation from the extracellular space is not possible. Thus, their connectivity to the network is attempted to be restored based solely on reversing directionalities, adding external reactions or adding transport reactions with the cytosol. In both single and multi-compartment models, downstream/upstream no-production/consumption metabolites are automatically fixed by restoring connectivity to their corresponding root no-production/consumption metabolite. Alternatively, we identify connectivity restoration mechanisms for downstream problem metabolites in addition to the indirect mechanisms through the fixing of root problem metabolites.

The proposed procedures are demonstrated on two widely used genome-scale metabolic models: *E. coli *[5] and *S. cerevisiae*[6]. The resultant connectivity resolving modifications in these models then serve as hypotheses. Specifically, we test if reactions can be reversed in the *E. coli *model (Mechanism 1) using two independent methods. First, we query the EcoCyc database [19] about the reversibility of the tested reaction and subsequently we examine the reaction free energy change ΔG values as approximated by Henry et al [20]. Values of ΔG close to zero are indicative of a reversible reaction because the sign of ΔG will be sensitive to small concentration changes in the participating metabolites. Given the limited information (compared to *E. coli*) available to thermodynamically characterize reactions in *S. cerevisiae*, we only employ the first method of testing for the latter model. We test if reactions are reversible by querying the MetaCyc database for corresponding information; if no such information is available, we check if the same reaction is reversible in other organisms.

Evidence for the presence of newly added reactions in the model is identified by checking for sequence similarity based on bidirectional BLAST scores searches [21]. Next, we determine whether a particular metabolite has an external uptake/secretion route (Mechanism 3) by searching for evidence in the open literature. Finally, in the case of multi-compartment models, we validate added intracellular transport reactions by examining whether metabolites with similar structures have known transport reactions in the metabolic network. The developed mathematical frameworks for identifying and filling gaps are discussed in the Methods section. The next two sections describe in detail the results obtained by applying the above procedures to the two most highly cited genome-scale models of *E. coli *[5] and *Saccharomyces cerevisiae *[6].

## Results

### *E. coli*

**Figure 4:**
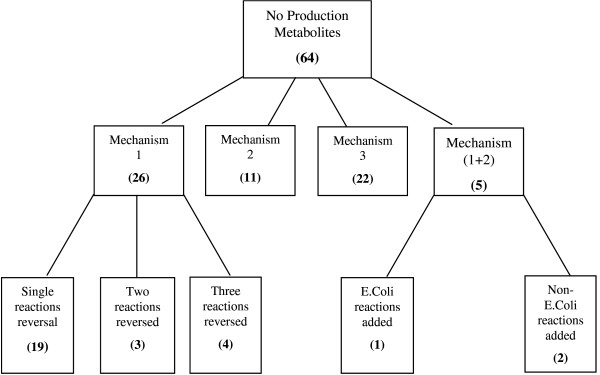
**Classification of mechanisms of restoring connectivity for no-production metabolites in *E. coli *using (GapFill)**. The numbers in the boxes indicate the mode of flow restoration for problem metabolites in *E. coli*.

In this study, we first identify all no-production metabolites (both root and downstream) using the (GapFind) formulation using the most recent *E. coli *genome-scale model [5]. All metabolites in the iJR904 model [5] that are identified as transport metabolites are allowed to enter and leave the cell. All metabolite and reaction abbreviations used in this section are taken from the iJR904 model [5]. Figure 4 summarizes the results obtained by using the (GapFind) formulation on the iJR904 model. As shown in Figure 4, there are 64 no-production metabolites; 28 of these metabolites are root and 36 are downstream no-production metabolites. Of the 64 identified no-production metabolites, 31.2% belong to Cofactor and Prosthetic Group Biosynthesis, 25% belong to Alternate Carbon Metabolism, 14% belong to Oxidative phosphorylation, 10.9% belong to Cell Envelope Biosynthesis, 7.8% belong to Nucleotide Salvage and the remaining 12.5% are not assigned to any pathway. The presence of so many unbalanced metabolites is quite surprising given how extensively this model has been curated and how widely it is used.

We next proceed with the gap-filling procedure using the (GapFill) formulation. We first identify the metabolites for which production mechanisms are established by reversing directionalities of existing reactions in *E. coli*. As shown in Figure 4, for 26 out of 64 no-production metabolites, production pathways are established by reversing directionality of existing reactions in *E. coli*. Also, 19 out of these 26 require a single reaction directionality reversal while three (i.e., 3dgulnp, 5prdmbz, adocbip, bbtcoa, ctbtcoa) require the reversal of directionality of two reactions while four metabolites (adocbi, dmbzid cbi and pc_EC) require the reversal of three reactions respectively. To ensure that all 26 metabolites are produced in the network, (GapFill) identifies that the directionalities of at least twenty eight reactions have to be reversed. It is important to note that the identified reaction directionality reversals leading to the establishment of production routes for the problematic metabolites are to a large extent unique. Specifically, only two additional reaction reversal are identified when the (GapFill) model is re-solved (using integer cuts) to exhaustively identify all possible reaction reversals capable of resolving all no-production metabolites (See Table 1). These results indicate that the production of most of the no-production metabolites can be enabled by expanding the directionality of existing reactions in *E. coli *rather than adding new ones.

**Table 1:** Reactions whose directions have to be reversed to restore connectivity of metabolites using mechanism 1.

**(GapFill) identified reversible reactions in *E. coli***	**Corresponding metabolite(s) in *E. coli *which is (are) fixed**	**Pathway**
**ADOCBLS**	rdmbzi, agdpcbi, adocbi, adocbip	Cofactor and Prosthetic Group Biosynthesis
**HETZK**	4mhetz	Cofactor and Prosthetic Group Biosynthesis
**HMPK1**	4ahmmp	Cofactor and Prosthetic Group Biosynthesis
**SPODM**	o2-	Unassigned
**DXYLK**	dxyl	Cofactor and Prosthetic Group Biosynthesis
**ACBIPGT**	adocbi, adocbip, 5prdmbz	Cofactor and Prosthetic Group Biosynthesis
**PGLYCP**	2pglyc	Alternate Carbon Metabolism
**NNDMBRT**	5prdmbz, dmbzid	Cofactor and Prosthetic Group Biosynthesis
**PEAMNO**	**peamn**	Alternate Carbon Metabolism
**ALDD19x**	**peamn**	Alternate Carbon Metabolism
CRNt7	crn, gbbtn, ctbt, crncoa*, bbtcoa, ctbtcoa	Transport, Extracellular
AP4AH	ap4a	Nucleotide Salvage Pathways
2DGLCNRy	2dhglcn	Alternate Carbon Metabolism
DKGLCNR2x	2dhglcn, 25dkglcn	Alternate Carbon Metabolism
BETALDHx	betald	Unassigned
BETALDHy	betald	Unassigned
DKGLCNR1	25dkglcn, 2dhglcn	Alternate Carbon Metabolism
X5PL3E	ap5a, xu5pL,	Alternate Carbon Metabolism
DKGLCNR2y	25dkglcn	Alternate Carbon Metabolism
DKGLCNR2x	25dkglcn, 2dhglcn	Alternate Carbon Metabolism
2DGULRx	2dhguln	Alternate Carbon Metabolism
2DGULRy	2dhguln	Alternate Carbon Metabolism
GP4GH	gp4g	Nucleotide Salvage Pathways
DOGULNR	23doguln, 3dhguln	Alternate Carbon Metabolism
ADOCBIK	adocbi	Cofactor and Prosthetic Group Biosynthesis
KG6PDC	3dgulnp, **3dhguln**	Alternate Carbon Metabolism
**RZ5PP**	dmbzid	Cofactor and Prosthetic Group Biosynthesis
GPDDA5	**g3pi**	Cell Envelope Biosynthesis
GPDDA3	g3ps	Cell Envelope Biosynthesis

The reactions in bold have support in the EcoCyc database. The pathways in which these reactions are present and the metabolites whose production they enable are as shown. The metabolites shown in bold are fixed by a combination of mechanisms 1 and 2.

The validity of the identified reaction directionality reversals is examined by employing two independent procedures as stated above. First we queried the identified reaction directionalities in the EcoCyc [19] database. We found that eleven out of the identified 30 reactions are listed as reversible in EcoCyc even though they are treated as irreversible in the iJR904 [5] model (Table 1). This provides independent verification for allowing reversing the directionality of these eleven reactions to be reversed when they are used in the context of the *E. coli *genome-scale model. As shown in Table 1 seven out of the eleven reactions belong to Cofactor and Prosthetic Group Biosynthesis, while two belong to Alternate Carbon Metabolism, one to Cell Envelope Biosynthesis and one is not assigned to any specific pathway. Notably, four of these eleven reactions which are treated as reversible in EcoCyc [19] (ADOCBLS, ACBIPGT, NNDMBRT and RZ5PP) are involved in enabling the production of more than one no-production metabolite. Of the remaining nineteen reactions for which the EcoCyc database does not provide positive evidence, the directionality of two reactions is unspecified whereas nine reactions take place in the direction specified in the iJR904 model. There is no information regarding the remaining eight reactions in the EcoCyc database.

**Figure 5:**
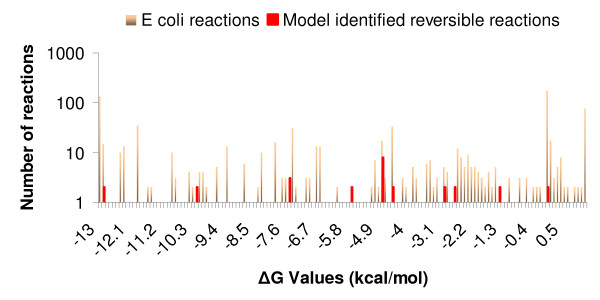
**ΔG values for (GapFill) identified reversible reactions in *E. coli***. Contrasting ΔG values between reactions found under Mechanism 1 and reactions in the iJR904 *E. coli *model on a log scale

**Figure 6:**
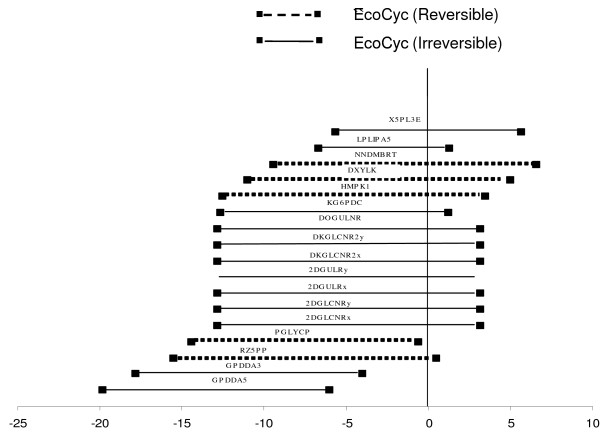
**Range of ΔG values for (GapFill) identified reversible reactions**. The abbreviations of the reactions are as shown on the horizontal lines. EcoCyc reversible reactions are those which have evidence in EcoCyc regarding their reversible nature, while EcoCyc irreversible reactions correspond to those that do not have any such evidence in EcoCyc.

As a second method of testing, we used the free energy change values ΔG of the identified reactions as approximated by Henry et al [20]. It should be noted here that ΔG values are available for only seventeen out of the 30 candidate reactions. It is likely that reactions that have calculated ΔG values closer to zero are reversible. These ΔG values are contrasted against the ΔG of all the reactions present in the *E. coli *iJR904 model according to the procedure of Henry et al [20] (see Figure 5). Upon quantifying the uncertainty in the approximated ΔG values based on the procedure by Henry and coworkers most of the identified reactions (fourteen out of seventeen) involve free energy ranges that span both negative and positive values (see Figure 6). This indicates that there is a reasonable likelihood that these fourteen reactions are reversible. Interestingly, five out of these fourteen reactions are also independently deemed as reversible based on the EcoCyc data (Figure 6). Using these two separate tracks of validation we find 35.71% unanimity in the prediction between the two methods.

**Figure 7:**
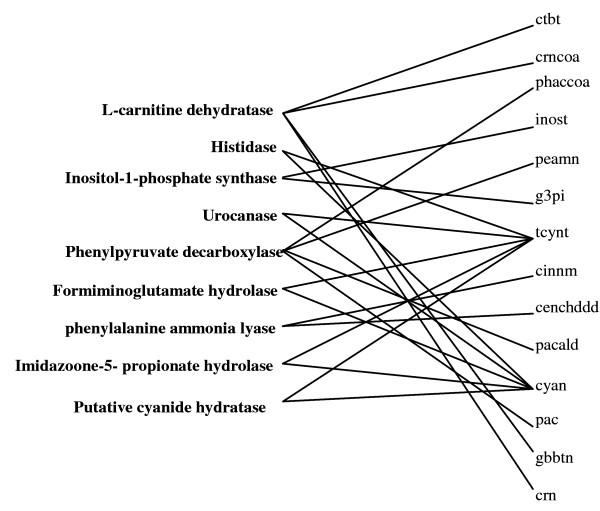
**Restoration of flow by adding missing reactions in *E. coli***. Mapping between the reactions (left hand side) added by (GapFill) for *E. coli *through mechanism 2 and the metabolites (right hand side) whose connectivity they (partially) serve to restore.

The second mechanism restores production routes by adding reactions from the database described in the previous section. As shown in Figure 4, eleven out of the 64 no-production metabolites are reconnected by adding reactions from the customized external database. The reactions and the corresponding metabolites are shown in Figure 7. (GapFill) identifies that at minimum nine reactions from the external database must be added. As seen from Figure 7, metabolites tcynt and cyan require the addition of the same set of five reactions from MetaCyc. Interestingly, two of these reactions L-carnitine dehydratase and putative cyanide hydratase are *E. coli *reactions that are present in the MetaCyc database but absent in the iJR904 model. Notably, the reaction putative cyanide hydratase is mentioned in [5] as a possible annotation for a conserved protein which is transcribed by the gene *ygiU*. For the remaining added reactions, we determine e-values obtained by checking for sequence similarity using the BLAST [21] algorithm between the candidate enzymes and the ORFs in the *E. coli *genome are shown in Table 2. The enzyme with the best bidirectional BLAST score of (1e-21,2e-23) phenylpyruvate decarboxylase, is involved in enabling the production of four no-production metabolites (Table 2). An e-value of 1e-21 indicates that only an expected number of 1e-21alignments with equivalent or better bit scores can occur in the database search by chance. The obtained low e-value four enzymes (see Table 2) are indicative that these functions may indeed be present in *E. coli*. Since the focus of this study is to provide testable hypotheses and not to conduct a thorough bioinformatics based analysis of missing enzymes, we believe that BLAST scores are sufficient. Alternatively, more elaborate scoring matrices based on Bayesian analysis and BLAST searches have been published by Green et al [11].

**Table 2:** BLAST scores of added reactions in *E. coli*

**Reactions**	**E Value**	**Best hit in *E. coli***	**Organism**
**formiminoglutamate hydrolase**	9e-13,3e-17	arginase/agmatinase/formimionoglutamate hydrolase	*Bacillus subtilis*
**Histidase**	3.8, 0.003	Putative formate acetyltransferase 3	*Bacillus subtilis*
**imidazolone-5-propionate hydrolase**	0.007,3e-07	guanine deaminase	*Bacillus subtilis*
**inositol-1-phosphate synthase**	no similarity	hypothetical protein EcolE_01002634	*Mycobacterium tuberculosis*
**phenylalanine ammonia lyase**	8.6, 3e-04	Ornithine/acetylornithine aminotransferase	*Arabidopsis thaliana*
**phenylpyruvate decarboxylase**	1e-21,2e-23	acetolactate synthase isozyme III	*Thauera aromatica*
**Urocanase**	0.006, 3e-07	hypothetical protein Z4243	*Bacillus subtilis*
**L-carnitine dehydratase**	------------	------------	*E. coli*
**putative cyanide hydratase**	------------	------------	*E. coli*

Candidate reactions from the MetaCyc database (along with BLAST scores) added to enable production of no-production metabolites in *E. coli*.

The connectivity restoration for five out of the sixty no-production metabolites requires the combination of Mechanisms 1 and 2 (see Figure 4). Specifically, for two of these metabolites (peamn and g3pi), in addition to requiring the reversal of directionality of existing reactions in the iJR904 model (see Table 1) additional reactions need to be added from MetaCyc to enable their production. Interestingly for metabolite 3dhguln the added reaction L-xylulose kinase is present in EcoCyc but absent in the iJR904 model [5].

The production of the remaining twenty two no-production metabolites (Figure 4) is possible only by the uptake of the corresponding metabolite from outside the cell. Four of these twenty four metabolites (dms, dmso, tma and tmao) even though they have an extracellular component in the iJR904 model, there are no corresponding reactions which explicitly allow transport into the cell (i.e., no reactions of the form metabolite A [e] --> A [c]). Due to their presence as extracellular components, it is reasonable to assume that the corresponding transport reactions may also be present. For the remaining twenty metabolites, the validity of adding uptake routes is tested by searching for corresponding evidence in literature. While no direct evidence was retrieved for the existence of uptake reactions for any of these sixteen metabolites, there exists evidence that *trans*-acontitate is formed spontaneously from *cis*-acontitate which is an intermediate in the citric acid cycle [22,23]. Note that all the hypotheses generated to fill gaps are available in an additional file [see Additional File 1]. In the next section we describe the results obtained by applying the (GapFill) and the (GapFind) procedures to the genome-scale model of *Saccharomyces cerevisiae *[6].

### *S. cerevisiae*

**Figure 8:**
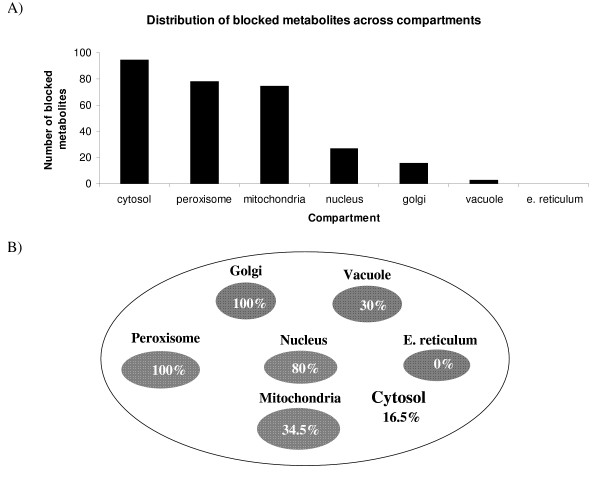
**Problem metabolites identified by (GapFind) in the *Saccharomyces cerevisiae *iND750 model**. 8A) shows the number of problem metabolites in each of the compartments. 8B) shows the percentage of metabolites in each of the compartments that are disconnected.

**Figure 9:**
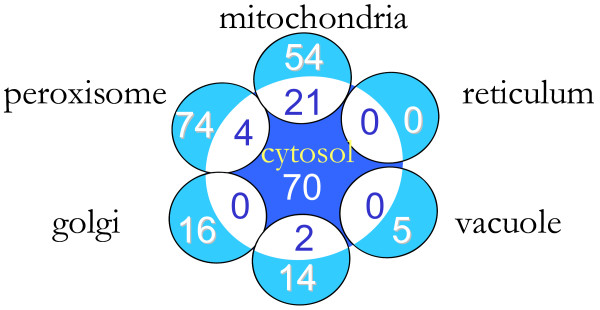
**Common problem metabolites between the cytosol and the inner compartments**. The numbers show the common problem metabolites between the cytosol and the remaining compartments.

In this study, we first identify the no-production metabolites using the modified form of (GapFind) for multi-compartment models in the *Saccharomyces cerevisiae *iND750 model [6]. All metabolite and reaction abbreviations used in this section are taken from the iND750 model [6]. Figure 8 shows the distribution of no-production metabolites across the different compartments in the *Saccharomyces cerevisiae *iND750 model. As shown in Figure 8(A), a majority of the problem metabolites are in the cytosol, the mitochondria and the peroxisome. Surprisingly, Figure 8(B) reveals that none of the metabolites in either the peroxisome or the golgi apparatus are accessible. On the other hand, all the metabolites in the endoplasmic reticulum are connected. Also, as shown in Figure 9, there are a number of common problem metabolites between the cytosol and the inner compartments. Notably, as shown in the figure, more than 25% of the problem metabolites in the mitochondria also cannot be produced in the cytosol. This suggests that identifying production pathways for cytosolic no-production metabolites may automatically fix some of the corresponding downstream problem metabolites in the inner compartments. Taking this into account, we restore flow through problem metabolites in two steps: First we identify production mechanisms for cytosolic no-production metabolites. Subsequently, the identified additions/modifications that fix cytosolic no-production metabolites are appended to the original network and the problem metabolites in the remaining compartments are identified using (GapFill) for each compartment separately.

**Figure 10:**
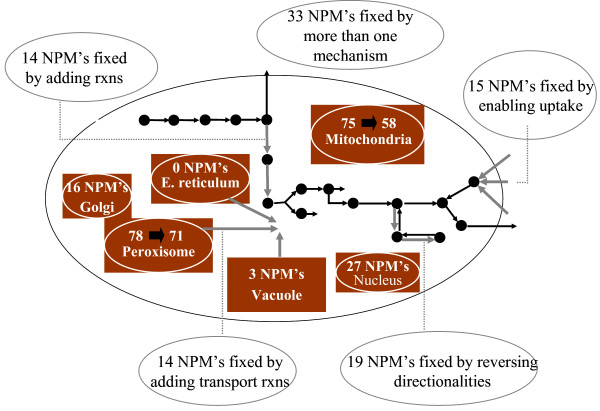
**(GapFill)-cytosol**. Production mechanisms identified by (GapFill) for cytosolic no-production metabolites. As shown, this results in the automatic fixing of seven peroxisomal and seventeen mitochondrial metabolites.

Figure 10 shows the distribution of the production mechanisms identified by (GapFill) to enable production of cytosolic no-production metabolites. As shown, a majority, i.e., 14 metabolites are fixed by adding transport reactions from other compartments to the cytosol. Also 14 metabolites are fixed by adding missing reactions from the MetaCyc database and 19 metabolites are fixed by reversing the directionalities of existing reactions in the *Saccharomyces cerevisiae *model. Interestingly, 33 no-production metabolites are fixed by more than one of the above mechanisms. Finally, since (GapFill) does not identify production mechanisms for any of the remaining fifteen no-production metabolites, we enable their production by enabling transport reactions for them from the environment. As shown in Figure 10, production pathways for seven peroxisomal and seventeen mitochondrial metabolites are automatically identified by fixing cytosolic no-production metabolites. Thus, identifying production pathways for cytosolic metabolites restores connectivity to 24 out of the 199 non-cytosolic problem metabolites.

**Figure 11:**
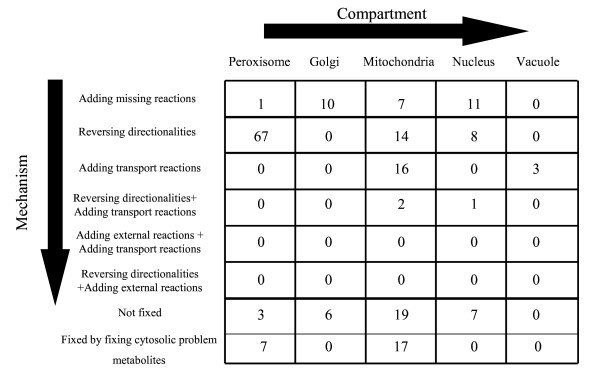
**(GapFill)-inner compartments**. Generated hypotheses identified by (GapFill) to fix problem metabolites in the inner compartments.

Connectivity restoration mechanisms for the remaining problem metabolites in the inner compartments are next identified using (GapFill). Figure 11 shows in detail the generated hypotheses to enable production of the remaining problem metabolites in non-cytosolic compartments. As shown a majority of the metabolites are fixed by reversing directionalities of existing reactions in the *Saccharomyces cerevisiae *model. Also, as shown in Figure 11, a large number of metabolites are fixed adding missing reactions from the MetaCyc database. However, it should also be noted that (GapFill) cannot identify production mechanisms for about 17.5% of all metabolites in the inner compartments. This means that none of these metabolites can be fixed by adding missing reactions from the MetaCyc database, reversing the directionalities of existing reactions in the model or adding intracellular transport reactions between the cytosol and the other compartments. Resolving these remaining inconsistencies would require currently absent functionalities and/or metabolites in the multi-species reaction database.

**Figure 12:**
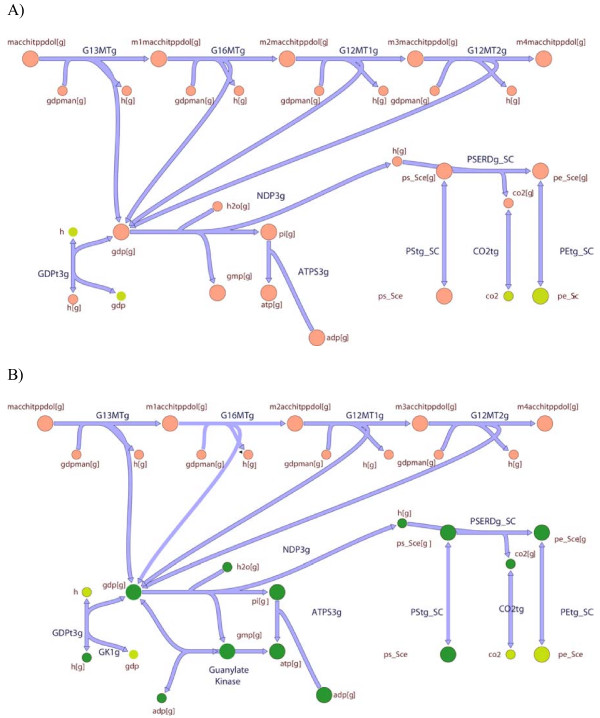
**The metabolic map of the Golgi apparatus**. (GapFind) identifies sixteen no-production metabolites, ten of which are resolved by (GapFill) by adding one reaction to the existing metabolic network as shown. The metabolites colored orange are not fixed, the ones colored dark green are fixed and the ones colored light green are cytosolic metabolites.

As an example, we examine in detail the results obtained for the golgi apparatus. As shown in Figure 12(A), (GapFind) identifies that the lack of flow in the root no-production metabolite, macchitppdol, results in fifteen downstream problem metabolites. (GapFill) does not identify a production mechanism for macchitppdol which would automatically enable production of the remaining fifteen metabolites. Instead, it fixes ten of the sixteen no-production metabolites by adding a reaction which is downstream of macchitppdol as shown in Figure 12(B). Interestingly, the enzyme guanylate kinase that catalyzes the added reaction is present in the cytosol where it catalyzes the same reaction. This information alludes to the possible presence of this activity in the golgi apparatus.

As shown in Figures 10 and 11, 144 (108 exclusively by reversing directionalities and 36 by a combination of reversing directionalities and other mechanisms) (36.7%) of the problem metabolites across all compartments are fixed by reversing the directionalities of 33 reactions in the *Saccharomyces cerevisiae *model. Ten out of the 33 reactions are reversible in other organisms according to information in the MetaCyc [18] database, four of them are always irreversible, seven have unspecified directionality in other organisms and twelve do not even have any information about the presence or absence of the corresponding enzyme in MetaCyc. Interestingly reversing the directionalies of FAO80p, a reaction that oxidizes Octanoyl-CoA as part of the fatty acid degradation pathway in the peroxisome, and FA80tp, a reaction that transports octanoate into the peroxisome from the cytosol, fixes 83% of all problem metabolites in the peroxisome. Notably, FAO80p is treated as reversible in other organisms in MetaCyc. A detailed list of hypotheses generated through (GapFill) are provided in the supplementary material [see Additional File 2].

Fifteen reactions are added to the existing *Saccharomyces cerevisiae *model to enable the production of 47 problem metabolites (43 exclusively by adding reactions and 4 by a combination of adding missing reactions and other mechanisms). Of these, 18 are cytosolic metabolites (Figure 10) and 29 are non-cytosolic metabolites (Figure 11)) metabolites the model. Table 3 shows the e-values obtained by checking for sequence similarity between the candidate enzymes and the ORF's in the *Saccharomyces cerevisiae *genome by performing the bidirectional BLAST analysis. As shown, eight of these enzymes have e-values less than 10^-13 ^in both the forward and reverse directions. This is indicative that these candidate enzymes and the corresponding best hits in *Saccharomyces cerevisiae *genome are orthologs and not paralogs [24]. Notably, four of these eight enzymes that fix non cytosolic metabolites are already present in the *Saccharomyces cerevisiae *genome as cytosolic reactions. This means that, in addition to identifying missing reactions in the metabolic network, (GapFill) predicts potential activities of existing enzymes across compartments in the model and hence, it could be used effectively to aid in deciphering additional potential locations for activities of existing enzymes in a genome-scale metabolic reconstruction.

**Table 3:** BLAST scores of added reactions in *S. cerevisiae*

**Reactions**	**E Value**	**Reaction in *S. cerevisiae***	**Organism**
**methionine &gamma;-lyase**	1e-58,1e-68	Cystathionine gamma-lyase	*Pseudomonas putida*
**2-hydroxyglutarate synthase(no gene identified yet)**	----------	---------------	*E. coli*
**2-oxoglutarate reductase/phosphoglycerate dehydrogenase**	2e-14, 3e-18	3-phosphoglycerate dehydrogenase	*Bacillus subtilis*
**deoxyribose-phosphate aldolase**	7.7, 0.006	Phosphatidylinositol 3-kinase TOR2	*Mycoplasma pneumoniae*
**D-lactate dehydrogenase**	2, 3e-04	Protein of unknown function	*E. coli*
**L-tryptophan aminotransferase**	no information	no information	*Enterobacter cloacae*
**Methylglyoxal synthase**	1.5, 0.001	carbamyl phosphate synthetase	*E. coli*
**phenylpyruvate decarboxylase**	3E-35, 1e-39	Chain A, Pyruvate Decarboyxlase	*Azoarcus sp. EbN1*
**tagatose-1,6-bisphosphate aldolase 2**	1e-12, 1e-16	Fructose 1,6-bisphosphate aldolase	*E. coli*
**tryptophan 2-monooxygenase**	2.2, 5e-05	orf:PZA105	*E. coli*
**5,10-methylenetetrahydrofolate reductase(present in the cytosol)**	1E-38, 2e-42	Isozyme of methylenetetrahydrofolate reductase	*E. coli*
**adenine phosphoribosyltransferase(present in cytosol)**	2E-25, 1e-29	Adenine phosphoribosyltransferase	*E. coli*
**guanylate kinase(present in cytosol)**	9E-38, 7e-42	Guanylate kinase	*E. coli*
**putative NAD+ kinase(present in cytosol)**	2E-27, 6e-28	ATP-NADH kinase	*E. coli*
**adenylate kinase(present in cytosol and mitochondria)**	7E-51, 5e-55	Adenylate kinase	*E. coli*

Candidate reactions from the MetaCyc database (along with BLAST scores) added to enable production of no-production metabolites in *S. cerevisiae*.

The production of the 65 (33 exclusively and 32 in combination with other mechanisms) problem metabolites in the *Saccharomyces cerevisiae *model is enabled by adding 22 intracellular transport reactions between the cytosol and the remaining compartments (Figures 10 and 11). The 43 cytosolic problem metabolites are fixed atleast partially (Figure 10) by adding 5 transport reactions from the other compartments; specifically three transport reactions between mitochondria and the cytosol (transporting the metabolites acACP, ACP, and malACP) and two transport reaction between the peroxisome and the cytosol (ttccoa, hexccoa transport). Examination of the prevalent transport mechanisms in the *Saccharomyces cerevisiae *model reveals that eight different fatty-acyl carrier protein groups can be transported between the cytosol and the mitochondria. Taking into consideration the structural similarity between different acyl carrier proteins, we can hypothesize that the three added intracellular transport reactions between the cytosol and the mitochondria transporting ACP, malACP and acACP are likely to be present in the *Saccharomyces cerevisiae *model. Also, because there are seven reactions that transport metabolites with Coenzyme A groups from the cytosol to the peroxisome, it is reasonable to hypothesize that the transport reactions transporting ttccoa and hexccoa between the peroxisome and the cytosol may indeed be present. Moving to the mitochondria, fifteen transport reactions added between the cytosol and the mitochondria serve to atleast partially (taking into account metabolites fixed by more that one mechanism) fix 16 problem mitochondrial metabolites. Two of these transport reactions serve to transport gdp and gtp between the cytosol and the mitochondria. Also, the three problem metabolites in the vacuole are fixed by adding transport reactions to transport adp and atp from the cytosol and the vacuole. Finally the production of fifteen cytosolic metabolites is enabled by uptaking them directly from the extracellular space. We found no evidence in the literature to support or contradict these uptake mechanisms.

## Discussion and conclusion

In this paper, we introduced two optimization based procedures, (GapFind) and (GapFill), to identify and fill gaps in genome-scale metabolic reconstructions. This was achieved by pinpointing metabolites can cannot be produced or consumed in the network using (GapFind) and then using (GapFill) to generate hypotheses that restored flow through these metabolites. These procedures were demonstrated on the single compartment model of *E. coli *and the multi-compartment model of *Saccharomyces cerevisiae*[6]. When applied to the single compartment model of *E. coli*, the (GapFind) procedure identified that about 10.4% of all metabolites were disjoint from the rest of metabolism. Flow through a majority of these metabolites was restored by reversing directionalities of existing reactions in the *E. coli *model. As many as 40% of them could not be fixed by any of the postulated mechanisms instead requiring their free uptake from the extracellular space. Flow through the remaining metabolites was restored by either exclusively adding missing reactions from MetaCyc or a combination of reversing directionalities of existing reactions in the model and adding missing reactions. Interestingly, for almost 50% of the reactions identified as reversible by (GapFill) had supporting evidence in the information obtained from the EcoCyc database. In the case of the multi-compartment model of *Saccharomyces cerevisiae*, (GapFind) identified that approximately 30% of all metabolites in the model were disconnected. Flow through 22% of them was restored by adding intracellular transport reactions in the model. Connectivity in the remaining 78% of the metabolites was restored by a combination of the mechanisms discussed in the case of *E. coli*. This left approximately 17.5% of all metabolites in the *Saccharomyces cerevisiae *model whose connectivity restoration cannot be accomplished though the postulated mechanisms.

For both models we found that a substantial percentage of the metabolites are disconnected from metabolism and cannot carry any flux. Despite these gaps *in silico *growth predictions using models of *E. coli *and *S. cerevisiae *are typically in good agreement with *in vivo *results [6,25]. This is primarily due to the fact that none of the identified gaps are in the well characterized central metabolism pathways and thus have no effect on growth prediction results. However, we anticipate that in *de novo *metabolic reconstructions of less well curated microorganisms using software applications such as the hole filler algorithm in the Pathway Tools software [26] gaps in central parts of metabolism are likely to be present leading to erroneous *in silico *predictions. We believe that for such automated reconstructions of less well studied microorganisms the utility of (GapFind) and (GapFill) will be even more pronounced. We have already applied some of these concepts in the reconstruction of the metabolic model of *Mycoplasma genitalium *currently underway in our group.

Clearly, the role of (GapFill) is to simply pinpoint a number of hypotheses which need to subsequently be tested. Using the basic set of our validation test we were able to relatively confidently validate or invalidate approximately 53% of the reversal of directionality of reactions that were predicted by (GapFill). An increase in experimental data, such as more information regarding ΔG values of reactions, which would help determine more accurately the thermodynamic feasibilities of transformations, will help increase this percentage. Also, use of recently developed computational procedures which combines available ΔG values with heuristic rules to elucidate thermodynamic constraints in genome-scale models [27] may also further sharpen the elucidation of correctness of the generated hypotheses. Furthermore, moving beyond the bidirectional BLAST hits that we used to validate newly added reactions in this study, the likelihood of the presence of the added enzymes can be more accurately assessed by using previously developed cost functions [8-10]. Also, by adopting concepts first proposed by Reed and co-workers [17], the gap-filled model can further be refined by contrasting *in silico *predictions of growth phenotypes with experimental observations. An inherent limitation of (GapFill) is the reliance on a candidate database of reactions. One could envision extending (GapFill) to consider hypothetical reactions in the spirit of the methods proposed by Hatzimanikatis and coworkers [28]. In conclusion, here, we introduced a systematic procedure to identify and fill gaps in metabolic reconstructions. As seen by the results obtained, these procedures can be used to curate existing metabolic reconstructions. In the future, we plan to deploy these methods during the generation phase of metabolic reconstructions of less curated microorganisms.

## Methods

### (GapFind): Identification of no-production metabolites

First we describe a straightforward procedure to identify root no-production metabolites in the metabolic network under steady-state conditions. Under steady-state conditions, metabolite balances for a metabolic network comprised of *M *metabolic reactions and *N *metabolites yield:

∑j∈MSijvj=bi∀i=1...N
 MathType@MTEF@5@5@+=feaafiart1ev1aaatCvAUfKttLearuWrP9MDH5MBPbIqV92AaeXatLxBI9gBaebbnrfifHhDYfgasaacH8akY=wiFfYdH8Gipec8Eeeu0xXdbba9frFj0=OqFfea0dXdd9vqai=hGuQ8kuc9pgc9s8qqaq=dirpe0xb9q8qiLsFr0=vr0=vr0dc8meaabaqaciaacaGaaeqabaqabeGadaaakeaafaqabeqacaaabaWaaabuaeaacqWGtbWudaWgaaWcbaGaemyAaKMaemOAaOgabeaakiabdAha2naaBaaaleaacqWGQbGAaeqaaOGaeyypa0JaemOyai2aaSbaaSqaaiabdMgaPbqabaaabaGaemOAaOMaeyicI4Saemyta0eabeqdcqGHris5aaGcbaGaeyiaIiIaemyAaKMaeyypa0JaeGymaeJaeiOla4IaeiOla4IaeiOla4IaemOta4eaaaaa@45D0@

where *b*_*i *_is a parameter which signifies if metabolite *i *is uptaken (*b*_*i *_is negative) or secreted (*b*_*i *_is positive), *v*_*j *_is the flux in reaction *j *of the metabolic network, *S*_*ij *_is the stoichiometric coefficient of metabolite *i *in reaction *j*. A metabolite *i *is inferred to be a root no-production metabolite by simply scanning the *i*^*th *^column of the matrix containing the *S*_*ij *_elements and examining whether there exist any positive entries (for irreversible reactions) or non-zero (for reversible reactions) signifying production/uptake terms. If no such entries are present then there is neither a direct production nor importation route for the tested metabolite implying that it forms the root node of a no-production branch (see Figure 1a). Note that root no-consumption metabolites can be identified in the metabolic network by scanning the stoichiometric matrix and employing a procedure symmetric to the one that identifies root no-production metabolites.

In single compartment models, the identification of downstream no-production metabolites cannot be accomplished by simply inspecting the entries of the *S*_*ij *_matrix. Instead, an optimization procedure is proposed to pinpoint downstream no-production metabolites. It is assumed that for all cytosolic metabolites a consumption term is assured because of the diluting effect of cell division acting as a metabolite sink, consumption by non-DNA controlled reactions, participation in macromolecular processes absent from the model involved in protein and DNA formation or simply export based on simple diffusion through the cell membrane. To incorporate this assumption, constraint (1) is rewritten as

∑j∈MSijvj≥0∀i=1...N
 MathType@MTEF@5@5@+=feaafiart1ev1aaatCvAUfKttLearuWrP9MDH5MBPbIqV92AaeXatLxBI9gBaebbnrfifHhDYfgasaacH8akY=wiFfYdH8Gipec8Eeeu0xXdbba9frFj0=OqFfea0dXdd9vqai=hGuQ8kuc9pgc9s8qqaq=dirpe0xb9q8qiLsFr0=vr0=vr0dc8meaabaqaciaacaGaaeqabaqabeGadaaakeaafaqabeqacaaabaWaaabuaeaacqWGtbWudaWgaaWcbaGaemyAaKMaemOAaOgabeaakiabdAha2naaBaaaleaacqWGQbGAaeqaaOGaeyyzImRaeGimaadaleaacqWGQbGAcqGHiiIZcqWGnbqtaeqaniabggHiLdaakeaacqGHaiIicqWGPbqAcqGH9aqpcqaIXaqmcqGGUaGlcqGGUaGlcqGGUaGlcqWGobGtaaaaaa@44B5@

The identification of the no-production metabolite set *NP *(a subset of *N*) requires first the introduction of binary variables *x*^*np*^_*i *_and *w*_*ij *_which are defined as:



Given a parent metabolic network consisting of a reaction set *M *and a set *N *of metabolites, the solution of the following optimization formulation (GapFind) identifies all downstream no-production metabolites in addition to the root no-production metabolites:



s.t

*S*_*ij*_*v*_*j *_≥ *εw*_*ij*_   ∀ *i *∈ *N*, *j*|(*S*_*ij *_> 0 *and j *∈ *IR*)

*S*_*ij*_*v*_*j *_≤ *Mw*_*ij*_   ∀ *i *∈ *N*, *j*|(*S*_*ij *_> 0 *and j *∈ *IR*)

*S*_*ij*_*v*_*j *_≥ *ε *- *M*(1 - *w*_*ij*_)   ∀ *i *∈ *N*, *j*|(*S*_*ij *_≠ 0 *and j *∈ *R*)

*S*_*ij*"_*v*_*j *_≥ *Mw*_*ij*_   ∀ *i *∈ *N*, *j*|(*S*_*ij *_≠ 0 *and j *∈ *R*)

*LB*_*j *_≤ *v*_*j *_≤ *UB*_*j*_, *j *∈ Model

∑jSijvj≥0∀i∈N
 MathType@MTEF@5@5@+=feaafiart1ev1aaatCvAUfKttLearuWrP9MDH5MBPbIqV92AaeXatLxBI9gBaebbnrfifHhDYfgasaacH8akY=wiFfYdH8Gipec8Eeeu0xXdbba9frFj0=OqFfea0dXdd9vqai=hGuQ8kuc9pgc9s8qqaq=dirpe0xb9q8qiLsFr0=vr0=vr0dc8meaabaqaciaacaGaaeqabaqabeGadaaakeaafaqabeqacaaabaWaaabuaeaacqWGtbWudaWgaaWcbaGaemyAaKMaemOAaOgabeaakiabdAha2naaBaaaleaacqWGQbGAaeqaaaqaaiabdQgaQbqab0GaeyyeIuoakiabgwMiZkabicdaWaqaaiabgcGiIiabdMgaPjabgIGiolabd6eaobaaaaa@3EDB@

*x*^*np*^_*i *_∈ {0, 1}, ∀*i*

*w*_*ij *_∈ {0, 1}, ∀*i*, *j*

{*j*' ∈ *M*|(*S*_*ij *_> 0 and *j *∈ *IR*) or (*S*_*ij *_≠ 0 *and j *∈ *R*)}

In (GapFind), sets *R *and *IR *comprise of reversible and irreversible reactions in the model respectively. Constraints (4) and (5) ensure that for each irreversible reaction that produces the metabolite *i*, the binary variable *w*_*ij *_assumes a value of one only if the reaction produces atleast *ε *units of metabolite *i *(i.e., *S*_*ij*_*v*_*j *_≥ 0). Similarly, constraints (6) and (7) ensure that for each reversible reaction in which the metabolite *i *participates, the binary variable *w*_*ij *_assumes a value of one only if the reaction produces *ε *units of metabolite *i*. Constraint (8) then ensures that metabolite *i *must have at least one production route if *x*^*np*^_*i *_= 1 by requiring a minimum of that atleast for one reaction *j *that can produce metabolite *i*, the value of a *w*_*ij *_= 1 consequently implying that atleast *ε *units of the metabolite *i *is produced. Constraint (9) restricts the fluxes in the reactions present in the model organism between predetermined upper and lower bounds, *UB*_*j *_and *LB*_*j *_(*LB*_*j *_= 0 for irreversible reactions). The net positive balance assumption which led to (2) is incorporated in constraint (10). Finally the objective function (3) maximizes the sum of the binary variables *x*^*np*^_*i *_over all metabolites ensuring the identification of all metabolites that have at least one production route given a set of available substrates. Therefore, if at the optimal solution *x*^*np*^_*i *_is equal to zero then metabolite *i *is a no-production metabolite thus belonging to set *NP*. By using this procedure, all no-production metabolites are identified. Metabolites identified previously as root no-production are subsequently subtracted from the list to yield only the downstream no-production metabolites.

The (GapFind) procedure is slightly modified for multi-compartment models. As shown in Figure 3, cytosolic metabolites can be drained out into the extracellular space while non-cytosolic metabolites can only be transported out into the cytosol. This implies that for metabolites present in compartments the production term must match exactly the consumption term (see constraint (12)) as they cannot freely drain into the extracellular space. This implies that the balance constraint (10) in (GapFind) is replaced by the following two constraints in multi-compartment models.

∑jSijvj≥0 ∀ i∈cytosol
 MathType@MTEF@5@5@+=feaafiart1ev1aaatCvAUfKttLearuWrP9MDH5MBPbIqV92AaeXatLxBI9gBaebbnrfifHhDYfgasaacH8akY=wiFfYdH8Gipec8Eeeu0xXdbba9frFj0=OqFfea0dXdd9vqai=hGuQ8kuc9pgc9s8qqaq=dirpe0xb9q8qiLsFr0=vr0=vr0dc8meaabaqaciaacaGaaeqabaqabeGadaaakeaadaaeqbqaaiabdofatnaaBaaaleaacqWGPbqAcqWGQbGAaeqaaOGaemODay3aaSbaaSqaaiabdQgaQbqabaGccqGHLjYScqaIWaamaSqaaiabdQgaQbqab0GaeyyeIuoakiabbccaGiabgcGiIiabbccaGiabdMgaPjabgIGiolabdogaJjabdMha5jabdsha0jabd+gaVjabdohaZjabd+gaVjabdYgaSbaa@4925@

∑jSijvj=0 ∀ i∉cytosol
 MathType@MTEF@5@5@+=feaafiart1ev1aaatCvAUfKttLearuWrP9MDH5MBPbIqV92AaeXatLxBI9gBaebbnrfifHhDYfgasaacH8akY=wiFfYdH8Gipec8Eeeu0xXdbba9frFj0=OqFfea0dXdd9vqai=hGuQ8kuc9pgc9s8qqaq=dirpe0xb9q8qiLsFr0=vr0=vr0dc8meaabaqaciaacaGaaeqabaqabeGadaaakeaadaaeqbqaaiabdofatnaaBaaaleaacqWGPbqAcqWGQbGAaeqaaOGaemODay3aaSbaaSqaaiabdQgaQbqabaaabaGaemOAaOgabeqdcqGHris5aOGaeyypa0JaeGimaaJaeeiiaaIaeyiaIiIaeeiiaaIaemyAaKMaeyycI8Saem4yamMaemyEaKNaemiDaqNaem4Ba8Maem4CamNaem4Ba8MaemiBaWgaaa@4852@

Note that a GAMS implementation of (GapFind) is available as an additional file [see Additional file 3].

### (GapFill): Gap resolution based on minimal metabolic model modifications

Upon the identification of all no-production metabolites in the model the next step involves filling these gaps using minimally the three mechanisms described earlier. We first explore whether reaction directionality reversal and/or addition of reactions from Metacyc [18] absent from the original model links the problem metabolite with the present substrates. This is accomplished by using a database of candidate reactions consisting of (i) all reactions in the original model with their directionalities reversed and (ii) reactions from a curated version of the MetaCyc database including allowable transport mechanism entries between compartments (in the case of multi-compartment models). It should be noted here that all the reactions in the MetaCyc database are treated as reversible in the model. These reactions define set *Database *comprised of candidate reactions while set *Model *is composed of the original genome-scale model reactions. It should be noted that if none of the above two/three mechanisms is capable of connecting the cytosolic no-production metabolite in single/multi-compartment models then an uptake reaction is arbitrarily added to the model to restore connectivity. However, if a non-cytosolic metabolite (in the case of multi-compartment models) present in an inner compartment cannot be fixed by any of the above mechanisms it is flagged as unfixable given the employed mechanisms.

In addition to the binary variable *w*_*ij *_defined previously, the proposed (GapFill) formulation relies on the binary variables *y*_*j *_defined as follows:

yj={1if reaction j from the external database is added to the parent network0otherwise
 MathType@MTEF@5@5@+=feaafiart1ev1aaatCvAUfKttLearuWrP9MDH5MBPbIqV92AaeXatLxBI9gBaebbnrfifHhDYfgasaacH8akY=wiFfYdH8Gipec8Eeeu0xXdbba9frFj0=OqFfea0dXdd9vqai=hGuQ8kuc9pgc9s8qqaq=dirpe0xb9q8qiLsFr0=vr0=vr0dc8meaabaqaciaacaGaaeqabaqabeGadaaakeaacqWG5bqEdaWgaaWcbaGaemOAaOgabeaakiabg2da9maaceqabaqbaeaabiGaaaqaaiabigdaXaqaaiabbMgaPjabbAgaMjabbccaGiabbkhaYjabbwgaLjabbggaHjabbogaJjabbsha0jabbMgaPjabb+gaVjabb6gaUjabbccaGiabdQgaQjabbccaGiabbAgaMjabbkhaYjabb+gaVjabb2gaTjabbccaGiabbsha0jabbIgaOjabbwgaLjabbccaGiabbwgaLjabbIha4jabbsha0jabbwgaLjabbkhaYjabb6gaUjabbggaHjabbYgaSjabbccaGiabbsgaKjabbggaHjabbsha0jabbggaHjabbkgaIjabbggaHjabbohaZjabbwgaLjabbccaGiabbMgaPjabbohaZjabbccaGiabbggaHjabbsgaKjabbsgaKjabbwgaLjabbsgaKjabbccaGiabbsha0jabb+gaVjabbccaGiabbsha0jabbIgaOjabbwgaLjabbccaGiabbchaWjabbggaHjabbkhaYjabbwgaLjabb6gaUjabbsha0jabbccaGiabb6gaUjabbwgaLjabbsha0jabbEha3jabb+gaVjabbkhaYjabbUgaRbqaaiabicdaWaqaaiabb+gaVjabbsha0jabbIgaOjabbwgaLjabbkhaYjabbEha3jabbMgaPjabbohaZjabbwgaLbaaaiaawUhaaaaa@99F0@

For the case of single compartment models, the task of identifying the minimal set of additional reactions that enable the production of a no-production metabolite i* is posed as the following mixed integer linear programming problem (GapFill).

Minimize∑j∈Databaseyj(GapFill)
 MathType@MTEF@5@5@+=feaafiart1ev1aaatCvAUfKttLearuWrP9MDH5MBPbIqV92AaeXatLxBI9gBaebbnrfifHhDYfgasaacH8akY=wiFfYdH8Gipec8Eeeu0xXdbba9frFj0=OqFfea0dXdd9vqai=hGuQ8kuc9pgc9s8qqaq=dirpe0xb9q8qiLsFr0=vr0=vr0dc8meaabaqaciaacaGaaeqabaqabeGadaaakeaafaqabeqacaaabaGaemyta0KaemyAaKMaemOBa4MaemyAaKMaemyBa0MaemyAaKMaemOEaONaemyzau2aaabuaeaacqWG5bqEdaWgaaWcbaGaemOAaOgabeaaaeaacqWGQbGAcqGHiiIZcqWGebarcqWGHbqycqWG0baDcqWGHbqycqWGIbGycqWGHbqycqWGZbWCcqWGLbqzaeqaniabggHiLdaakeaacqGGOaakcqqGhbWrcqqGHbqycqqGWbaCcqqGgbGrcqqGPbqAcqqGSbaBcqqGSbaBcqGGPaqkaaaaaa@549F@

s.t

*S*_i*j_*v*_*j *_≥ *δ *- *M*(1 - *w*_i*j_)   ∀ *j*|(*S*_*ij *_≠ 0)

*S*_i*j_*v*_*j *_≤ *Mw*_i*j_   ∀ *j*|(*S*_*ij *_≠ 0)

∑jwi∗j≥1
 MathType@MTEF@5@5@+=feaafiart1ev1aaatCvAUfKttLearuWrP9MDH5MBPbIqV92AaeXatLxBI9gBaebbnrfifHhDYfgasaacH8akY=wiFfYdH8Gipec8Eeeu0xXdbba9frFj0=OqFfea0dXdd9vqai=hGuQ8kuc9pgc9s8qqaq=dirpe0xb9q8qiLsFr0=vr0=vr0dc8meaabaqaciaacaGaaeqabaqabeGadaaakeaadaaeqbqaaiabdEha3naaBaaaleaacqWGPbqAcqGHxiIkcqWGQbGAaeqaaOGaeyyzImRaeGymaedaleaacqWGQbGAaeqaniabggHiLdaaaa@3836@

∑jSijvj≥0∀i∈N
 MathType@MTEF@5@5@+=feaafiart1ev1aaatCvAUfKttLearuWrP9MDH5MBPbIqV92AaeXatLxBI9gBaebbnrfifHhDYfgasaacH8akY=wiFfYdH8Gipec8Eeeu0xXdbba9frFj0=OqFfea0dXdd9vqai=hGuQ8kuc9pgc9s8qqaq=dirpe0xb9q8qiLsFr0=vr0=vr0dc8meaabaqaciaacaGaaeqabaqabeGadaaakeaafaqabeqacaaabaWaaabuaeaacqWGtbWudaWgaaWcbaGaemyAaKMaemOAaOgabeaakiabdAha2naaBaaaleaacqWGQbGAaeqaaOGaeyyzImRaeGimaadaleaacqWGQbGAaeqaniabggHiLdaakeaacqGHaiIicqWGPbqAcqGHiiIZcqWGobGtaaaaaa@3EF0@

*LB*_*j *_≤ *v*_*j *_≤ *UB*_*j*_,   ∀ *j *∈ Model

*LB*_*j*_·*y*_*j *_≤ *v*_*j *_≤ *UB*_*j*_·*y*_*j*_,   ∀ *j *∈ *Database*

*w*_*ij *_∈ {0, 1},   ∀*i*, *j*

*y*_*j *_∈ {0, 1},   ∀ *j *∈ *Database*

{*j*' ∈ *M *| (*S*_*ij *_≠ 0)}

In (GapFill), the objective function (13) minimizes the number of added reactions from the *Database *so as to restore flow through metabolite i*. Constraints (14) and (15) are identical to (6) and (7). Constraint (16) ensures that these additions are subject to a minimum of *δ *units for the no-production metabolite i* being produced. Constraint set (17), as in (GapFind), allows for the free drain of all cytosolic metabolites while bounds on reactions present in the *Model *are imposed by constraint set (18). Constraint set (19) ensures that only those reactions from the *Database *that have non zero flow are added to the model. This formulation restores flow through no-production metabolites in single compartment models. For multi-compartment models, the (GapFill) formulation is modified. First gaps in the cytosol are filled using the mechanisms described earlier for single compartment models. Specifically, the (GapFill) formulation is modified by replacing constraint (17) with constraints (11) and (12) reflecting the fact that no net production term can be imposed for metabolites present with compartments incapable of communicating directly with the extracellular space. The solution of formulation (GapFill) once for each no-production metabolite i* identifies one mechanism at a time for resolving connectivity problems in the model. It should be noted that through the use of integer cuts [29] multiple hypotheses can be generated to resolve these connectivity problems. In this study, we evaluate the merit of generated hypotheses and subsequently choose the most probable one using the following three criteria sequentially a) The added hypotheses should not have cycles: since the MetaCyc database consists of multiple copies of the same reaction (which are present in different organisms), there is a proclivity to fix metabolites by adding two copies of the same reaction in opposite directions (since all reactions in the MetaCyc database are considered reversible) thereby forming a cycle, b) We choose the hypotheses which enables production of the problem metabolite with the least number of modifications and c) We choose a hypotheses that has higher probability of being accurate based on our validation metrics (e.g., if two reactions are added, we choose the one with the better blast score). Note that a GAMS implementation of (GapFill) is available as an additional file [see Additional file 4].

## Authors' contributions

CDM conceived the study. VSK, MSD and CDM provided the mathematical formulations for (GapFind) and (GapFill). VSK implemented (GapFind) and (GapFill). VSK, MSD and CDM drafted the manuscript. All authors read and approved the final version of the manuscript.

## Supplementary Material

Additional file 1 **Testable hypotheses for *E. coli***. The abbreviations used are from the *E. coli *iJR904 model. Click here for file

Additional file 2 **Testable hypotheses for *S. cerevisiae***. The abbreviations used are from the *S. cerevisiae *iND750 model. Click here for file

Additional file 3 **(GapFind)**. The (GapFind) formulation in the GAMS modeling language. Click here for file

Additional file 4 **(GapFill)**. The (GapFill) formulation in the GAMS modeling language. Click here for file
